# Comparison of the effectiveness of thiamethoxam and its main metabolite clothianidin after foliar spraying and root irrigation to control *Myzus persicae* on peach

**DOI:** 10.1038/s41598-022-20659-w

**Published:** 2022-10-07

**Authors:** Fajun Tian, Chengkui Qiao, Caixia Wang, Tao Pang, Linlin Guo, Jun Li, Rongli Pang, Hui Liu, Hanzhong Xie

**Affiliations:** Zhengzhou Fruit Research Institute, Chinese Academy of Agricultural Sciences, Zhengzhou, 450009 China

**Keywords:** Analytical chemistry, Mass spectrometry, Chemical biology

## Abstract

The green peach aphid, *Myzus persicae*, is one of the most economically important pests in peach-growing areas around the world. In many countries, the application of insecticides is the main method to control and reduce the population of *M. persicae*. In this study, we investigated the effects and persistence of thiamethoxam against *M. persicae* by foliar spraying and root irrigation. The residues of thiamethoxam and clothianidin in peach were determined to assess food safety. The results showed that thiamethoxam treatment significantly reduced the population of *M. persicae* through foliar spraying and root irrigation. And the persistence of root irrigation on *M. persicae* was significantly longer than that of spraying. Thiamethoxam and clothianidin were absorbed by the roots, transported to other parts of the plant, and concentrated in the leaves, especially new leaves. The final residues of thiamethoxam and clothianidin in peaches were below the maximum residue limit (MRLs). These results suggested that thiamethoxam is more effective in *M. persicae* control through root irrigation than foliar spraying. The persistence of root irrigation on *M. persicae* was significantly longer than that of spraying. These results shed some light upon the control of *M. persicae* by root irrigation of thiamethoxam.

## Introduction

The green peach aphid, *Myzus persicae* (Sulzer) (Hemiptera: Aphididae), is a serious pest in crop worldwide. This insect has a typical polyphagia and can cause major damage to more than 400 plant species^1^. *M. persicae* directly damages by feeding on sap from plant phloem and indirectly damages by transmitting over 100 plant viruses^2^. And it also produce large lots of honeydew, which ultimately causes growth of the fungus and a reduction in photosynthesis, causing significant damage to commercial crop production. Currently, the use of insecticides to control damage caused by *M. persicae* on many crops is the most effective strategy in many countries worldwide. Many countries use the method of foliar spraying of broad-spectrum insecticides. *M. persicae* feeds on sap from plant phloem on young leaves. Therefore, a large amount of pesticide spraying is required to obtain a satisfactory control effect. It has a short-term residual activity of 2–4 weeks^3^. Their intensive use has negative consequences, including destruction of the ecological environment, the decimation of non-target organisms, resulting in secondary pest outbreaks, pesticide drift and workers’ exposure^4^. Meanwhile, the synthetic insecticide has been used intensively over many years. *M. persicae* has developed significant resistance to more than 75 compounds, including organochlorines, organophosphates, carbamates, pyrethroids and neonicotinoids^5–7^. Therefore, it is important to explore a simple and economically feasible method to reduce or eliminate these adverse effects.

At present, root irrigation is a main mode of pesticide application and considered an effective management strategy for the control of *M. persicae*. Root irrigation is mainly the application of pesticides to the roots of plants^8^. After irrigation, the pesticide could be absorbed through the roots and then transported to the upper part of the plant by the xylem, causing pests to be poisoned or died after consumption of plants^9,10^. The advantages of irrigation pesticide application are that it can reduce the number of pesticide applications and its drift. It also provide long-lasting activity with minimal impact on non-target organisms^8,10^. Some studies have shown that the levels of some pesticides in young shoots were higher than in old shoots through root irrigation. Meanwhile, adults and nymphs of *M. persicae* are mainly found on young leaves. Therefore, root irrigation is considered an effective management method for the control of *M. persicae*.

Thiamethoxam (EZ)-3-(2-chloro-1,3-thiazol-5-ylmethyl)-5-methyl-1,3,5-oxadiazinan-4-ylidene(nitro) amine, was a second-generation neonicotinoid and introduced in 1998, which has the characteristics of high effect, low toxicity and strong systemic translocation^11,12^. Thiamethoxam acts as an agonist of nicotinic acetylcholine receptors (nAChRs) and is widely used to control sucking and biting insect pests in many crops, such as, *M. persicae*, *Trialeurodes vaporariorum* (Homoptera: Aleyrodidae) and *Diaphorina citri* Kuwayama (Hemiptera: Psyllidae),^13,14^. The commercial thiamethoxam have been registered on a global scale for foliar, root irrigation and seed treatment^10,15,16^. The previous report suggested that thiamethoxam was transformed to clothianidin in soils, insects, and plants after application^17^. Because clothianidin has a higher affinity for insect nAChRs than thiamethoxam, it has higher insecticidal activity than thiamethoxam^18^. Hence, the part of the insecticidal activity of thiamethoxam was related to clothianidin. However, some studies have found that thiamethoxam and clothianidin can cause rats to release more dopamine in vivo striatum^19^. Therefore, it is important to understand the fate of thiamethoxam and its metabolite clothianidin and correctly evaluate its safety. However, only few studies have been reported on the effectiveness and safety of thiamethoxam for root irrigation.

In the current study, we investigated the dynamic changes of *M. persicae* control by root irrigation and foliar spray of thiamethoxam. In addition, thiamethoxam and clothianidin concentrations in leaves were quantified to evaluate the efficacy against *M. persicae*. Meanwhile, the residual status in peach fruits were also determined. The results of this study provide more accurate information for *M. persicae* pest control.

## Results

### Effect of thiamethoxam applied by foliar spraying and root irrigation on *M. persicae*

The control effect of thiamethoxam spraying on *M. persicae* was better in the early stage, and the control effect on *M. persicae* was more than 84.1% after 1–7 days. Meanwhile, the control effect increased significantly with the increase of the application concentration. With the prolongation of time, the control effect decreased, showing poor “persistence”. The control effect in the low-concentration spray began to decline 5 days after treatment, and dropped to 65.4% at 14 days, and only 28.5% at 30 days. Although the control effect of high concentration spray was better than that of low concentration, the control effect also began to decline 7 days after the treatment, and the control effect was only 42.3% at 30 days. The results showed that the control effect of the foliar spraying application lasted for 7–10 days and decreased rapidly after 14 days (Table 1). The application of thiamethoxam by root irrigation has poor control effect in the early stage. Root irrigation of thiamethoxam at 8 g tree^−1^ only resulted in a control of 50.3% after 3 days. However, the control effect of low- and high-concentration treatment is over 80% after 7 days. The control effect of low- and high-concentration root irrigation treatments reached 93.3% and 97.5% after 21 days of application, respectively. After 30 days, the control effect of low- and high-concentration root irrigation remained above 60%. Therefore, the persistence of root irrigation on *M. persicae* was significantly longer than that of spraying.

**Table 1:** Control effect of thiamethoxam at low and high concentrations on *Myzus persicae* under two application methods.

Application method	Dosage	Control effect (mean (%) ± SE)					
		1 day	3 days	7 days	14 days	21 days	30 days
Foliar spraying	30 g a.i./ha	84.1 ± 3.4 a	91.1 ± 1.4 a	92.8 ± 3.6 a	65.4 ± 1.9 a	45.6 ± 1.8 a	28.5 ± 1.1 a
	60 g a.i./ha	94.6 ± 2.6 b	97.4 ± 1.7 a	96.3 ± 1.5 a	78.6 ± 2.7 b	63.1 ± 0.9 b	42.3 ± 0.6 b
Root irrigation	4 g tree^−1^	14.7 ± 1.6 c	41.4 ± 2.1 b	80.7 ± 3.4 b	89.5 ± 1.5 c	93.3 ± 1.2 c	65.4 ± 1.3 c
	8 g tree^−1^	21.4 ± 1.3 c	50.3 ± 2.4 b	85.7 ± 2.1 b	93.7 ± 2.9 c	97.5 ± 0.8 c	84.1 ± 2.7 d

Values are means ± SE of the three replicates.

Note: The means within each rank followed by different letters are significantly different at *p* < 0.05 (Tukey’s test).

### Method validation

Seven-point calibration curves for thiamethoxam and clothianidin in matrix solution (peach and leaves) from 1, 10, 50, 100, 500, 1000, and 5000 μg kg^−1^ were constructed. Outstanding linearities of the calibration curves were obtained for each compound (R^2^ ≥ 0.9989) in peach and leaves (Table 2). The mean recoveries of thiamethoxam and clothianidin at five spiking levels with five replicates in peaches and leaves are shown in Table 3. The mean recoveries of thiamethoxam and clothianidin were within 81.3–110.7%, and the RSD values were ranged from 0.9 to 8.1%. The LOQ for thiamethoxam and clothianidin in different matrices was defined as the minimum spiked concentration with acceptable recovery (70–120%) and precision (RSD ≤ 20%)^20^. The LOQ values of thiamethoxam and clothianidin in leaves and peaches were 5 and 1 μg kg^−1^, respectively (Table 2). These results suggested that the proposed method was reliable for the determination of thiamethoxam and clothianidin.

**Table 2:** Calibration equations, R^2^ and LOQ.

Compounds	Matrix	Regression equation	R^2^	LOQs (μg kg^−1^)
Thiamethoxam	Leaves	y = 159.8x + 5376.3	0.9991	5
	Peach	y = 2937.3x + 84308.6	0.9996	1
Clothianidin	Leaves	y = 167.1x + 18744.2	0.9993	5
	Peach	y = 2475.8x + 153894.8	0.9989	1

**Table 3:** Recoveries and RSDs of thiamethoxam and clothianidin in peaches samples at different fortification levels (n = 5).

Matrix	Spiked level (μg kg^−1^)	Thiamethoxam		Clothianidin	
		Recovery (%)	RSD (%)	Recovery (%)	RSD (%)
Leaves	5	109.5	4.3	104.3	1.8
	10	110.3	1.9	110.7	6.9
	100	88.3	2.9	86.7	5.9
	1000	81.3	2.0	84.8	3.1
	5000	83.3	5.7	88.7	5.0
Peach	1	98.0	5.3	102.8	1.8
	10	95.7	2.7	95.5	1.9
	100	89.9	7.9	92.9	8.1
	1000	84.9	4.4	86.8	2.5
	5000	88.1	2.5	89.1	0.9

### Dissipation of thiamethoxam and clothianidin in peach and leaves

**Figure 1:**
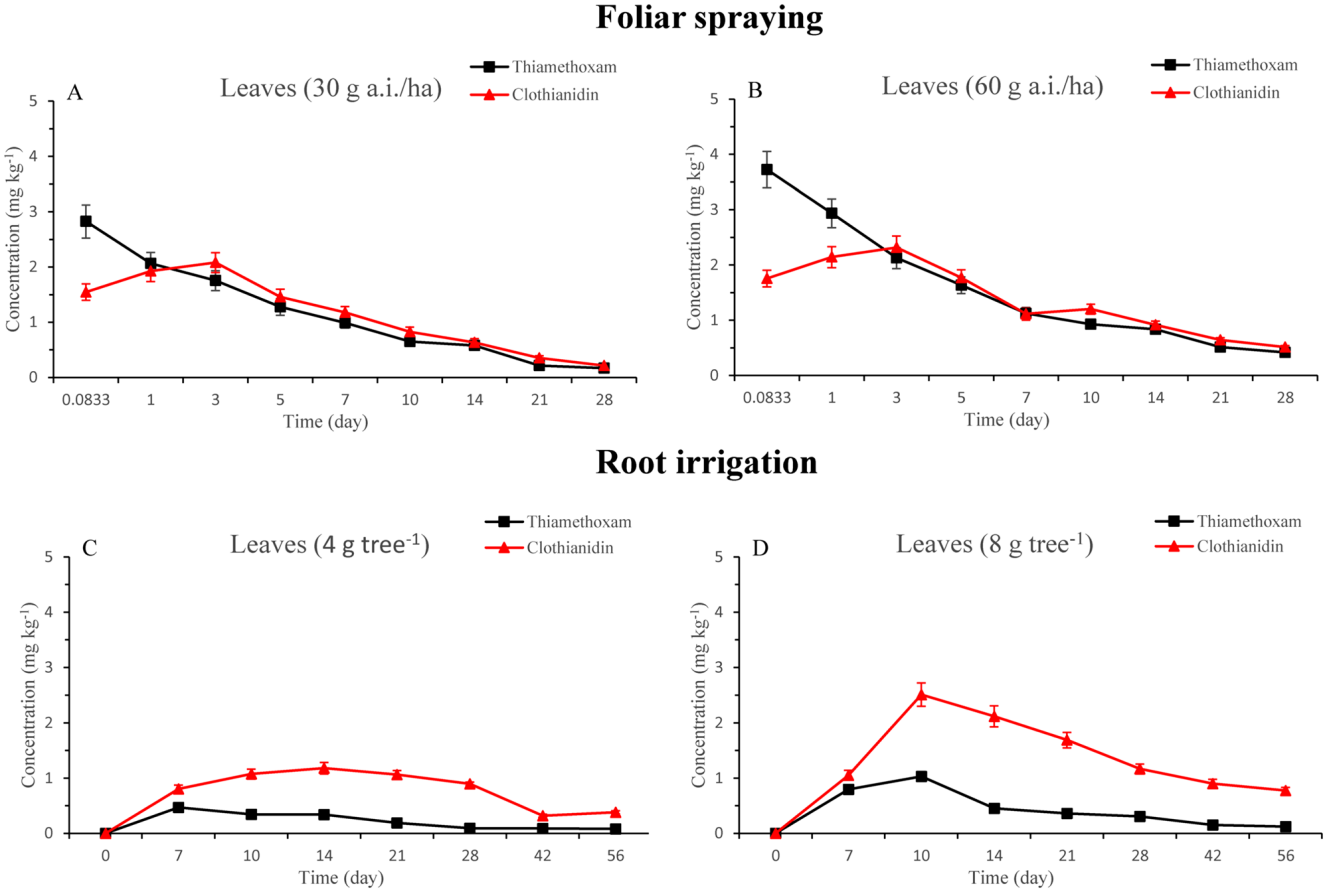
Residues of thiamethoxam and clothianidin in leaves after application by foliar spraying at standard (**A**) and 2 times (**B**) dose treatments and by root irrigation at 4 g tree^−1^ (**C**) and 8 g tree^−1^ (**D**).

**Figure 2:**
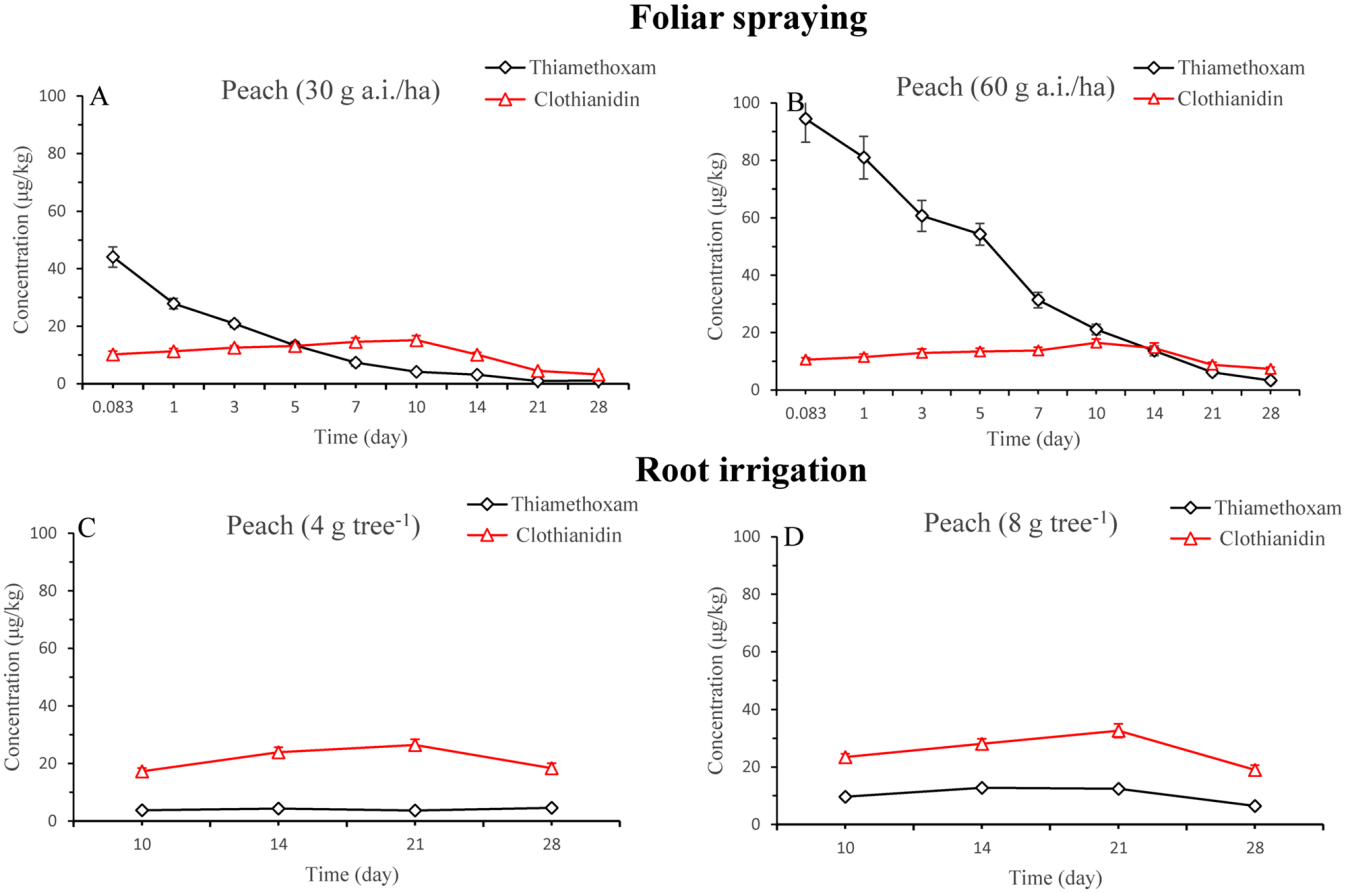
Residues of thiamethoxam and clothianidin in peaches after application by foliar spraying at standard (**A**) and 2 times (**B**) dose treatments and by root irrigation at 4 g tree^−1^ (**C**) and 8 g tree^−1^ (**D**).

The dissipation pattern of thiamethoxam and degradation product clothianidin in peaches and leaves were shown in Figs. 1 and 2. The dissipation equations and half-life values (t_1/2_) were shown in Table 4 by foliar spraying. The results indicated that thiamethoxam is transformed to clothianidin in soils. Meanwhile, thiamethoxam and clothianidin were also absorbed by peach roots and accumulated in leaves, and then thiamethoxam was metabolized to clothianidin. In peaches and leaves, the concentrations of thiamethoxam and clothianidin were higher in the high-concentration treatment than in the low-concentration treatment. For foliar spraying, the thiamethoxam residues were decreased with time elapse in peaches and leaves. And the dissipation followed the first-order kinetic (Table 4). When thiamethoxam was applied at doses of 30 and 60 g a.i./ha (gram of active ingredient per hectare), the respective concentrations of thiamethoxam after 2 h were 0.044 and 0.094 mg kg^−1^ and 2.82 and 3.72 mg kg^−1^ in the peaches and leaves, respectively. The amount of thiamethoxam dissipated by 97.6 and 96.8% and 94.1and 88.8% on day 28 in the peaches and leaves, respectively. When thiamethoxam was applied at low-concentration, the residues were dissipated to an extent of 52.7 and 54.9% after 3 days in the peaches and leaves showing residues of 0.021 and 1.75 mg kg^−1^. Following that period, the residues of thiamethoxam in peaches and leaves decreased by 90.7 and 77.1% after 10 days, respectively. However, when thiamethoxam was applied at high-concentration, the residues were dissipated to an extent of 52.3 and 56.2% after 5 days in the peaches and leaves showing residues of 0.045 and 1.63 mg kg^−1^. Following that period, the residues of thiamethoxam in peaches and leaves decreased by 86.2 and 77.7% after 14 days, respectively. When thiamethoxam was applied at low- and high-concentration, the half-lives of thiamethoxam in peaches and leaves were 4.95 and 5.58 days and 6.79 and 9.24 days, respectively (Table 4). For root irrigation, the concentrations of thiamethoxam in leaves increased from 0 to 10 days, peaking at 0.47 and 1.03 mg kg^−1^ at low- and high-concentration treatments, respectively. Then, the concentrations decreased with the time elapse. In the low-concentration treatment, thiamethoxam reached plateau faster than in the high-concentration.

**Table 4:** Regression equations, half-lives (T_1/2_) and correlation coefficients (R^2^) for the dissipation of thiamethoxam at different application dosage by foliar spraying.

Dosage	Compounds	Sample	Regression equation	R^2^	T_1/2_ (day)
Recommended application dosage	Thiamethoxam	Leaves	C_t_ = 2.25e^−0.102t^	0.9690	6.79
Two-fold recommended application dosage	Thiamethoxam	Leaves	C_t_ = 2.62e^−0.075t^	0.9027	9.24
Recommended application dosage	Thiamethoxam	Peaches	C_t_ = 27.13e^−0.140t^	0.9129	4.95
Two-fold recommended application dosage	Thiamethoxam	Peaches	C_t_ = 83.58e^−0.124t^	0.9913	5.58

Clothianidin, the major metabolite of thiamethoxam, was formed in peaches and leaves under field conditions. Figures. 1 and 2 presented the residues of clothianidin in the peaches and leaves at different doses treatments. The amount of initially increased and then decreased with time elapse. For foliar spraying, the residue of clothianidin in peaches was highest at 10 days and the concentrations were 0.015 and 0.016 mg kg^−1^ at low- and high-concentration treatments, respectively. The initial amount of clothianidin was 0.01 and 0.011 mg kg^−1^ from low- and high-concentration treatments. Approximately 12.5 and 8.0% of thiamethoxam metabolized to clothianidin at 10 days from low- and high-concentration treatments after application. The amounts of clothianidin were 0.0032 and 0.0073 mg kg^−1^ in peaches at 28 days after application at low- and high-concentration treatments, respectively. However, the residue of clothianidin in leaves was highest at 3 days and the concentrations were 2.08 and 2.31 mg kg^−1^ at low- and high-concentration treatments, respectively. The initial amount of clothianidin was 1.55 and 1.75 mg kg^−1^ from low- and high-concentration treatments. Approximately 49.9 and 35.2% of thiamethoxam metabolized to clothianidin at 3 days from low- and high-concentration treatments after application. The amounts of clothianidin were 0.21 and 0.51 mg kg^−1^ in leaves at 28 days after application at low- and high-concentration treatments, respectively. For root irrigation, the highest concentrations of clothianidin in leaves were found at 14 and 10 days after application at 4 and 8 g tree^−1^, peaking at 1.18 and 2.51 mg kg^−1^, respectively. The concentrations then decreased with the time elapse (Fig. 1). In the 56 days after application, the amounts of clothianidin were 0.038 and 0.77 mg kg^−1^ in leaves at 4 and 8 g tree^−1^, respectively. The highest concentrations of clothianidin in peaches were found at 21 days after different treatments, peaking at 0.026 and 0.033 mg kg^−1^, respectively. Then, the amounts of clothianidin decreased with the time elapse.

### Thiamethoxam and clothianidin concentration in new and old leaves

In the 30 days after application, the amounts of thiamethoxam and clothianidin in new and old leaves at 8 g tree^−1^ were detected. Accumulated thiamethoxam and clothianidin in new leaves were higher than those in the old leaves. The amounts of thiamethoxam and clothianidin in new leaves were 0.39 and 2.13 mg kg^−1^, respectively. The amounts of thiamethoxam and clothianidin in old leaves were 0.21 and 0.85 mg kg^−1^, respectively. The amounts of thiamethoxam and clothianidin in new leaves at 8 g tree^−1^ treatment was 1.86 and 2.51 times higher than those in old leaves, respectively. Meanwhile, adults and nymphs of *M. persicae* are mainly found on new leaves. Therefore, the feature is more beneficial to control aphids *M. persicae*.

### Residues of thiamethoxam and clothianidin in peaches

Figure 2 presented the residues of thiamethoxam and clothianidin in the peach by different treatments (foliar spraying and root irrigation) at different doses. For foliar spraying, the maximum amounts of thiamethoxam and clothianidin in peach were 0.094 and 0.015 mg kg^−1^ after 2 h of its application at the two-fold recommended dosage, respectively. For root irrigation, the residues of thiamethoxam and clothianidin in peach were highest after 21 days of its application at 8 g tree^−1^, and the amounts of thiamethoxam and clothianidin were 0.012 and 0.033 mg kg^−1^, respectively. The residues of thiamethoxam and clothianidin in peach was much below the maximum residue limit (MRLs) required by the China (thiamethoxam, 1 mg kg^−1^; clothianidin 0.2 mg kg^−1^).

## Discussion

In China, controlling *M. persicae* by spraying insecticides is the common strategy^21^. This study compared the effectiveness of thiamethoxam application via foliar spraying and root irrigation to control *M. persicae* on peach. The results indicated that thiamethoxam via root irrigation was more effective in controlling *M. persicae* than foliar spraying. Foliar application resulted in a 90% reduction in control of *M. persicae* within 7 days compared with controls. However, thiamethoxam was transported to peaches and leaves by systemic function was low. Therefore, the lasting validity period of spraying was shorter than that of root irrigation. Besides, root irrigation indicated a longer lasting validity period, reduced repeated use of pesticides, and has a higher control effect on *M. persicae*. The results were consistent with previous studies that thiamethoxam had a good control effect by root irrigation. Li et al.^22^ found that the control effect of thiamethoxam was the highest 10 days after root irrigation, reaching 94. 3%, and the effective period lasted for 20 days in cucumber. *M. persicae* is a sucking pest preferentially ingests phloem sap from young shoots rather than old shoots. A study by Li et al.^23^ proved that thiamethoxam via root irrigation mainly transported to the upper part of the tree and young shoots of plants by the xylem. We also found that the amounts of thiamethoxam and its main metabolite clothianidin were higher in new leaves than in old leaves. The exact timing of pesticide application is critical for effective pest control. *M. persicae* has about 20 generations a year and the generations overlap. Farmers need to spray big amount of pesticide in the production process. However, excessive use of pesticides could lead to the development of insecticide resistance, pesticide residues, environmental pollution and ecological balance disruption^24^. The peach trees produce a large amount of new flushes per season. This facilitates the continuous transport of thiamethoxam from the roots through xylem to the new growth, providing longer protection against *M. persicae*.

Some studies also found that root irrigation of pesticides can effectively control pests^8,10,25^. Thiamethoxam can be absorbed through the roots after root irrigation and then quickly transported to other parts of the plant by the xylem. There are many factors that affect the uptake and accumulation of pesticides in plants, such as root activity, temperature, soil type and rainfall et al.^26^. A study by Langdon et al. proved that the higher levels of thiamethoxam in the leaf tissue of small trees than in large trees were due to the fact that the small trees are actively growing^27^. Root growth was affected by shoot growth, soil water content and temperature. The root system began to expand in February, with the strongest root growth occurring in June and July. During our experiment, adequate development of the root system increased the uptake of thiamethoxam. Peach fruits required a lot of water and nutrients to grow and accumulate sugar. In addition, stronger transpiration promotes water absorption summer. These two features facilitated the absorption of thiamethoxam and clothianidin following water and nutrients. The concentrations of thiamethoxam and clothianidin were highest in the leaves. It may be that thiamethoxam was absorbed from the roots, transported upward and then stored in the leaves. And the concentration of clothianidin were higher than thiamethoxam, likely, because thiamethoxam was metabolized to clothianidin in peach tree. At 1 week after the application to the root, the amounts of thiamethoxam and clothianidin remained high, implying that thiamethoxam and clothianidin were continuously absorbed through the roots and transported to other parts of the plant by the xylem. The amounts of thiamethoxam and clothianidin in new leaves were higher than those in the old leaves. It may be that the life activity of new leaves are vigorous and the metabolism was fast. In addition, it has a fast conduction speed and large conduction amount. Therefore, the accumulated thiamethoxam and clothianidin in new leaves were significantly higher than those in the old leaves. Thiamethoxam was absorbed by roots and converted to clothianidin. The amount of clothianidin was higher than thiamethoxam. Clothianidin represented high activity against sucking pests, including *M. persicae*, and was even more than thiamethoxam^21,28^. Benzidane et al.^29^ found that toxicity of thiamethoxam on locomotion activity of cockroaches was related to its metabolite clothianidin. The accumulated thiamethoxam and clothianidin remained higher at 50 days after root irrigation, which could kill nymphs in time, reduce the population density of *M. persicae*, and improve the control effect. Root irrigation could effectively control *M. persicae* for 30 days compared with foliar spraying for 7 days. The sustainability of thiamethoxam and clothianidin within leaves minimized problems from foliar spraying, and the pesticide application process had little effect on non-target organisms and application time.

It may be that thiamethoxam and clothianidin were transported into peaches due to their systemic properties. The final residues of thiamethoxam and clothianidin were determined in peaches to evaluate food safety. For foliar spraying, the residues of thiamethoxam and clothianidin in peaches ranged from 0.094 to 0.00091 mg kg^−1^ and 0.015 to 0.0032 mg kg^−1^ in both treatments, respectively. For root irrigation, the residues of thiamethoxam and clothianidin in peaches ranged from 0.012 to 0.0036 mg kg^−1^ and 0.033 to 0.017 mg kg^−1^ in both treatments, respectively. The residues of thiamethoxam and clothianidin were below the MRLs of 1 mg kg^−1^ for thiamethoxam and 0.2 mg kg^−1^ for clothianidin in peach established by China. These results showed that thiamethoxam via root irrigation does not cause food safety problems.

## Conclusions

This study was designed to investigate dynamic changes of *M. persicae* control by root irrigation and foliar spray of thiamethoxam. Root irrigation of peach with thiamethoxam was implied to control *M. persicae* more effectively than foliar spraying. And control periods were positively related to the persistence of thiamethoxam and its main metabolite clothianidin in leaves. Thiamethoxam and clothianidin were absorbed by the roots and mostly concentrated in the leaves, which also increased the insect mortality of *M. persicae* nymphs. This pest control method could reduce pesticide residues and safely and effectively control *M. persicae*. And thiamethoxam does not cause food safety problems through root irrigation. However, thiamethoxam and clothianidin may be transported into the pollen and nectar of peach through the xylem and have toxic effects on pollinators, and should be further researched. Therefore, thiamethoxam at 4 g tree^−1^ was applied by root irrigation in a low *M. persicae* population density or commercially managed peach orchards.

## Materials and methods

### Chemicals and reagents

Thiamethoxam and clothianidin standards (purity > 99%) were supplied by Dr. Ehrenstorfer (LGC Standards, Augsburg, Germany). Thiamethoxam (50% WG) was obtained from the Laoting Yoloo Bio-technology Co., Ltd. (Tangshan, China). Analytical grade sodium chloride (NaCl) and anhydrous magnesium sulfate (MgSO_4_) were purchased from Sinopharm Chemical Reagent Co., Ltd. (Beijing, China). Milli-Q water (Millipore, Milford, MA, USA) was used throughout this study. Chromatography grade acetonitrile was purchased from Honeywell International Inc. (New Jersey, USA). Primary secondary amine (PSA, 40 μm) sorbent and 0.22-μm nylon syringe filter were supplied by Agela Technologies (Tianjing, China).

Individual stock standard solutions of thiamethoxam (1000 mg mL^−1^) and clothianidin (1000 mg mL^−1^) in chromatography grade acetonitrile were prepared. Mixed standard solutions of thiamethoxam and clothianidin were diluted with acetonitrile to the 1–5000 μg L^−1^ during the experiments. The matrix-matched standard solutions (1–5000 μg L^−1^) were prepared by applying appropriate volumes of standard solutions to untreated leaf and peach. All standard solutions were kept at 4 °C until use.

### Field experiments

The field trials were carried out in Zhengzhou in the north central of the Henan Province, China (114.1 °E, 34.7 °N) during the 2021 agricultural season (May–July). All experimental designs contained three replicate plots and one control plot with six trees in each randomized complete block. Each plot was separated by two rows of peach trees. The experiments had five treatments: (1) 60 g/hectare (recommended dosage) thiamethoxam applied through foliar spraying; (2) 120 g/hectare (2 times of recommended dosage) thiamethoxam applied through foliar spraying; (3) 4 g/tree thiamethoxam applied through root irrigation; (4) 8 g/tree thiamethoxam applied through root irrigation; and (5) the control, which not use thiamethoxam and clothianidin treatment. Thiamethoxam solutions were irrigated into the root zone of each tree through 10 L plastic buckets. The thiamethoxam was applied on the peach using a calibrated Xinxiu 3WBS-D-16A battery-powered knapsack sprayer (Zhengzhou, China). Thiamethoxam WG was sprayed under windless and sunny conditions on fruit surfaces, leaves (both sides), and branches of the targeted peach trees until it formed droplets on the fruit surface and dripped. For spraying and irrigation, the number of *M. persicae* (including all instars) on five vigorous new shoots in each direction (north, south, east and west) of each three trees per treatment were counted at 1, 3, 7, 14, 21 and 30 days. At each sampling day, pest reduction rate (PRR) was calculated based on the observed mortality for each treatment. Afterwards, corrected mortality was calculated and expressed as the control effect (CE). The PRR and CE were calculated as follows:

1 $${\text{PRR }}\left( \% \right)\, = \,\left( {C\, - \,T} \right)/C\, \times \,{1}00\%$$

2 $${\text{CE }}\left( {\text{\% }} \right) = \left( {\frac{{{\text{PRR in treatment }}{-}{\text{ PRR in the control}}}}{{100 - {\text{ PRR in the control}}}}} \right) \times 100{\text{\% }}$$

where *C* and *T* are the number of *M. persicae* in the initial and after treatment, respectively.

### Field sample collection

Representative peach samples (3 kg) and leaves were collected from the experimental plots 0 (2 h after treatment), 1, 3, 5, 7, 10, 14, 21 and 28 days after foliar spraying. Leaves from treated trees were randomly collected at 7, 10, 14, 21, 28, 42 and 56 days after irrigation. Peaches were randomly collected at 10, 14, 21 and 28 days. Three replicates were collected for each sample. All the samples were transferred to the laboratory and kept at − 20 °C until further analysis.

### Sample extraction and purification

Thiamethoxam and clothianidin were extracted using the method of Tian et al.^15^ with modification. 5 g of thoroughly homogenized peach leaves and 10 g of thoroughly homogenized peach were weighted into a 50 mL Teflon centrifuge tube. 5 mL water (only for leaf sample) and 10 mL acetonitrile was added into each tube. Then, the tubes were shaken for 10 min. Afterward, NaCl (1 g) and anhydrous MgSO_4_ (4 g) were added to each tube. The tubes were shaken vigorously on the vortex for 5 min, then centrifuged at 2077 *g* for 5 min. 1.5 mL of the supernatant solution of each sample was transferred into the disposable centrifuge tube containing 50 mg PSA and 150 mg anhydrous MgSO_4_. The tubes were shaken vigorously using a vortex for 1 min and then centrifuged at 2400 *g* for 5 min. Finally, the supernatant was filtered through a 0.22 μm nylon syringe filter into an autosampler vial for analysis.

### Instrument conditions

Thiamethoxam and clothianidin were separated and determined by high-performance liquid chromatography coupled to tandem mass spectrometry (HPLC–MS/MS) (Agilent Technologies, Inc., Santa Clara, CA, USA). Thiamethoxam and clothianidin were separated by gradient elution using an Agilent Poroshell 120 EC-C18 column (2.1 × 100 mm, 2.7 μm) at 30 °C. The mobile phases were water (A) and acetonitrile (B). The flow rate was 0.3 mL min^−1^. The mobile phase gradient program was shown in Table 5. The injection volume was 5 μL. Thiamethoxam and clothianidin detection was operated using electrospray ionization in positive ionization mode. The capillary voltage was 4000 V. The drying gas and sheath gas temperature were both 350 °C. The drying gas and sheath gas flow rate was 10.0 and 12.0 L min^−1^, respectively. All MS parameters are presented in Table 6.

**Table 5:** Gradient condition of a mobile phase composed of two solutions.

Time/min	Water/%	Acetonitrile/%
0	70	30
1	70	30
2	60	40
3	10	90
4	70	30
5	70	30

**Table 6:** Analytical conditions of the target compounds.

Compound	Precursor ion	Fragmentor (V)	Product ion	Collision energy (V)	RT (min)
Thiamethoxam	292.2	70	210.8^a^	10	1.23
			180.9^b^	20	
Clothianidin	250.1	75	169.1^a^	10	1.48
			131.9^b^	15	

^a^Quantifier.

^b^Qualifier.

### Data analysis

All data were presented as means ± standard deviations of triplicate. Statistical significance (*P* < 0.05) was analyzed by Tukey’s test using the Statistical Package for Social Sciences (SPSS; version 19.0 for Windows). The dissipation dynamics of thiamethoxam and clothianidin were governed by the first-order kinetic equation C_t_ = C_0_*e*^−kt^ and the half-life (T1/2) was calculated as follows: t_1/2_ = ln2/k = 0.693/*k*^15^.

### Ethical approval

We have permission to collect peach plant from the land owner. Experimental research and field studies on plants were carried out in accordance with relevant guidelines and regulations.

## Data Availability

The datasets used and analysed during the current study are available from the corresponding author on reasonable request.

## References

[1] Troczka BJ (2021). Molecular innovations underlying resistance to nicotine and neonicotinoids in the aphid *Myzus persicae*. Pest Manag. Sci..

[2] Ma Z (2022). A first greenhouse application of bacteria-expressed and nanocarrier-delivered RNA pesticide for *Myzus persicae* control. J. Pest Sci..

[3] Papadimitriou F (2022). Flupyradifurone resistance in *Myzus persicae* populations from peach and tobacco in Greece. Pest Manag. Sci..

[4] He J (2018). Greenhouse and field-based studies on the distribution of dimethoate in cotton and its effect on *Tetranychus urticae* by drip irrigation. Pest Manag. Sci..

[5] Bass C (2014). The evolution of insecticide resistance in the peach potato aphid, *Myzus persicae*. Insect Biochem. Mol. Biol..

[6] Cai H, Yang L, Zuo Z, Liao W, Yang Z (2021). Resistance status of *Myzus persicae* to pesticide and its relationship with enzymes. Agron. J..

[7] Philippou D, Field L, Moores G (2010). Metabolic enzyme (s) confer imidacloprid resistance in a clone of *Myzus persicae* (Sulzer)(Hemiptera: Aphididae) from Greece. Pest Manag. Sci..

[8] Wu J (2021). Examination of acephate absorption, transport, and accumulation in maize after root irrigation for *Spodoptera frugiperda* control. Environ. Sci. Pollut. R..

[9] Tian F (2016). Simultaneous determination of penflufen and one metabolite in vegetables and cereals using a modified quick, easy, cheap, effective, rugged, and safe method and liquid chromatography coupled to tandem mass spectrometry. Food Chem..

[10] Hu W (2019). Uptake of soil-applied thiamethoxam in orange and its effect against Asian citrus psyllid in different seasons. Pest Manag. Sci..

[11] Ullah F (2020). Thiamethoxam induces transgenerational hormesis effects and alteration of genes expression in *Aphis gossypii*. Pestic. Biochem. Physiol..

[12] Tesovnik T (2020). Exposure of honey bee larvae to thiamethoxam and its interaction with *Nosema ceranae* infection in adult honey bees. Environ. Pollut..

[13] Tian F, Li C, Wang Z, Liu J, Zeng X (2018). Identification of detoxification genes in imidacloprid-resistant Asian citrus psyllid (Hemiptera: Lividae) and their expression patterns under stress of eight insecticides. Pest Manag. Sci..

[14] Hilton MJ, Jarvis TD, Ricketts DC (2016). The degradation rate of thiamethoxam in European field studies. Pest Manag. Sci..

[15] Tian F (2022). The fate of thiamethoxam and its main metabolite clothianidin in peaches and the wine-making process. Food Chem..

[16] Maienfisch P (2001). The discovery of thiamethoxam: A second-generation neonicotinoid. Pest Manag. Sci..

[17] Fan Y, Shi X (2017). Characterization of the metabolic transformation of thiamethoxam to clothianidin in *Helicoverpa armigera* larvae by SPE combined UPLC–MS/MS and its relationship with the toxicity of thiamethoxam to *Helicoverpa armigera* larvae. J. Chromatogr. B.

[18] Nauen R, Ebbinghaus-Kintscher U, Salgado VL, Kaussmann M (2003). Thiamethoxam is a neonicotinoid precursor converted to clothianidin in insects and plants. Pestic. Biochem. Physiol..

[19] de Oliveira IM, Nunes BVF, Barbosa DR, Pallares AM, Faro LRF (2010). Effects of the neonicotinoids thiametoxam and clothianidin on in vivo dopamine release in rat striatum. Toxicol. Lett..

[20] Tian F (2021). Development and validation of a method for the analysis of trifludimoxazin, picarbutrazox and pyraziflumid residues in cereals, vegetables and fruits using ultra-performance liquid chromatography/tandem mass spectrometry. Microchem. J..

[21] Wei X (2017). Cross-resistance pattern and basis of resistance in a thiamethoxam-resistant strain of *Aphis gossypii* Glover. Pestic. Biochem. Physiol..

[22] Xiuhuan L, Haina W, Quan Z, Wei M (2011). Control effect of five insecticides against *Bemisia tabaci* (Gennadius) on cucumber plants with root pouring. Pestic. Sci. Admin..

[23] Li Y, Xie Y, Xu H (2016). Comparing xylem mobility of four types of pesticides, glucose-fipronil conjugate, fipronil, thiamethoxam and abamectin, in soybean. J. South China Agric. Univ..

[24] Hu M (2021). Insight into the adsorption mechanisms of ionizable imidazolinone herbicides in sediments: Kinetics, adsorption model, and influencing factors. Chemosphere.

[25] Li X (2021). Drip application of chlorantraniliprole effectively controls invasive *Spodoptera frugiperda* (Lepidoptera: Noctuidae) and its distribution in maize in China. Crop Prot..

[26] Zhang P (2018). Dissipation and residue of clothianidin in granules and pesticide fertilizers used in cabbage and soil under field conditions. Environ. Sci. Pollut. R..

[27] Langdon K, Schumann R, Stelinski L, Rogers M (2018). Influence of tree size and application rate on expression of thiamethoxam in citrus and its efficacy against *Diaphorina citri* (Hemiptera: Liviidae). J. Econ. Entomol..

[28] Puinean AM (2013). Development of a high-throughput real-time PCR assay for the detection of the R81T mutation in the nicotinic acetylcholine receptor of neonicotinoid-resistant *Myzus persicae*. Pest Manag. Sci..

[29] Benzidane Y (2010). Effect of thiamethoxam on cockroach locomotor activity is associated with its metabolite clothianidin. Pest Manag. Sci..

